# Delivery room resuscitation intensity and associated neonatal outcomes of 24^+0^–31^+6^ weeks’ preterm infants in China: a retrospective cross-sectional study

**DOI:** 10.1007/s12519-023-00738-2

**Published:** 2023-06-30

**Authors:** Si-Lu Wang, Chun Chen, Xin-Yue Gu, Zhao-Qing Yin, Le Su, Si-Yuan Jiang, Yun Cao, Li-Zhong Du, Jian-Hua Sun, Jiang-Qin Liu, Chuan-Zhong Yang, Chinese Neonatal Network Investigators

**Affiliations:** 1 Department of Neonatology, Shanghai Key Laboratory of Maternal Fetal Medicine, Shanghai Institute of Maternal-Fetal Medicine and Gynecologic Oncology, Shanghai First Maternity and Infant Hospital, School of Medicine, Tongji University, No. 2699, Gaoke Western Road, Pudong District, Shanghai, 201204 China; 2 Department of Neonatology, Affiliated Shenzhen Maternity and Child Healthcare Hospital, Southern Medical University, No. 2004, Hongli Road, Futian District, Shenzhen, 518028 China; 3 NHC Key Laboratory of Neonatal Diseases, Fudan University, Children’s Hospital of Fudan University, Shanghai, China; 4 Department of Neonatology, People’s Hospital of Dehong, Kunming Medical University, Dehong, China; 5 Department of Neonatology, Children’s Hospital of Fudan University, Shanghai, China; 6 Department of Neonatology, Children’s Hospital, Zhejiang University School of Medicine, Hangzhou, China; 7 Department of Neonatology, Shanghai Children’s Medical Center, School of Medicine, Shanghai Jiao Tong University, Shanghai, China

**Keywords:** Cardiopulmonary resuscitation, Delivery room, Endotracheal intubation, Neonatal resuscitation, Preterm

## Abstract

**Background:**

The aim of this study was to review current delivery room (DR) resuscitation intensity in Chinese tertiary neonatal intensive care units and to investigate the association between DR resuscitation intensity and short-term outcomes in preterm infants born at 24^+0^–31^+6^ weeks’ gestation age (GA).

**Methods:**

This was a retrospective cross-sectional study. The source population was infants born at 24^+0^–31^+6^ weeks’ GA who were enrolled in the Chinese Neonatal Network 2019 cohort. Eligible infants were categorized into five groups: (1) regular care; (2) oxygen supplementation and/or continuous positive airway pressure (O_2_/CPAP); (3) mask ventilation; (4) endotracheal intubation; and (5) cardiopulmonary resuscitation (CPR). The association between DR resuscitation and short-term outcomes was evaluated by inverse propensity score-weighted logistic regression.

**Results:**

Of 7939 infants included in this cohort, 2419 (30.5%) received regular care, 1994 (25.1%) received O_2_/CPAP, 1436 (18.1%) received mask ventilation, 1769 (22.3%) received endotracheal intubation, and 321 (4.0%) received CPR in the DR. Advanced maternal age and maternal hypertension correlated with a higher need for resuscitation, and antenatal steroid use tended to be associated with a lower need for resuscitation (*P* < 0.001). Severe brain impairment increased significantly with increasing amounts of resuscitation in DR after adjusting for perinatal factors. Resuscitation strategies vary widely between centers, with over 50% of preterm infants in eight centers requiring higher intensity resuscitation.

**Conclusions:**

Increased intensity of DR interventions was associated with increased mortality and morbidities in very preterm infants in China. There is wide variation in resuscitative approaches across delivery centers, and ongoing quality improvement to standardize resuscitation practices is needed.

**Supplementary Information:**

The online version contains supplementary material available at 10.1007/s12519-023-00738-2.

## Introduction

The transition of the fetus from an intrauterine to extrauterine environment is accomplished through multiple physiological changes that occur before and at birth, and most newborns can successfully complete this transition without medical intervention. However, up to 10% of newborns will need some degree of resuscitation after birth, and approximately 5% of term infants receive positive-pressure ventilation (PPV) to make a successful transition, 2% are intubated, 0.1% receive cardiac compressions, and 0.05% receive compressions with epinephrine [1, 2]. Preterm infants generally need more resuscitation interventions at birth due to their anatomical and physiological characteristics, especially 6%–7% of preterm infants < 32 weeks’ gestational age (GA) [3]. High resuscitation intensity increases the risk of short- or long-term adverse outcomes, which is influenced by initial resuscitation in the first few minutes in the delivery room (DR) [1].

The Neonatal Resuscitation Program guidelines published by the American Academy of Pediatrics recommend a standardized set of resuscitation practices in DR, including initial steps, PPV, endotracheal intubation, cardiopulmonary resuscitation (CPR), and medications [4]. Most infants are stabilized with non-invasive respiratory support after birth, but about 40% of preterm infants still require higher-level resuscitation methods, such as endotracheal intubation for invasive respiratory support [5, 6].

In China, a study demonstrated a significant correlation between different treatment practices and tertiary hospitals in each study. The number of preterm infants is increasing in different provinces, and treatment practices are improving [7]. This means that there is variation in Chinese hospitals and a lack of adherence to standardized neonatal resuscitation guidelines based on the China neonatal resuscitation guideline [8]. However, there is still a gap in treatment practices compared to developed countries.

As the Chinese Neonatal Network (CHNN) is a large standardized collaborative network of 70 tertiary Chinese neonatal intensive care units (NICUs), this research aimed to describe the variations in the intensity of resuscitation in DR for preterm infants with a GA of 24^+0^–31^+6^ in China. The research also aimed to investigate the correlation between different highest levels of intensity and short-term outcomes of very preterm infants in DR.

## Methods

### The Chinese Neonatal Network and participating hospitals

The CHNN is a national network of Chinese tertiary NICUs with the primary goal of conducting high-quality collaborative research dedicated to the improvement of neonatal-perinatal health in China [9]. Hospitals enrolled in CHNN are required to be tertiary referral hospitals with large neonatal services and have expertise in caring for high-risk neonates. CHNN has established and maintained a standardized clinical database of preterm infants < 32 weeks’ gestation or < 1500 g with participating NICUs throughout China. This clinical database has been monitoring outcomes and care practices since January 1, 2019. A total of 57 hospitals in 25 provinces across the country collected data using the CHNN database in 2019. These 57 hospitals included four national children’s medical centers, four regional children’s medical centers, and 30 provincial perinatal or children’s medical centers. The other 19 hospitals comprised major referral centers in large cities across China. Forty-three hospitals were perinatal centers with birthing facilities, and 14 hospitals were free-standing children’s hospitals that only admitted outborn infants. All hospitals had the ability to provide care for infants < 32 weeks’ gestation.

### Study population

The study population included infants delivered between January 1, 2019 and December 31, 2019 at 24^+0_^31^+6^ weeks’ GA who were admitted to CHNN hospitals and were retrospectively reviewed. Infants admitted within 3 days of age were included in this study. Stillbirth, DR deaths, and transfers to non-participating hospitals within 24 hours after birth were not included in this analysis. Readmissions and transfers between participating hospitals were tracked as data from the same infants. Infants were followed until NICU discharge or transfer or death. Infants with major congenital anomalies, unknown resuscitation details, palliative care, or no active resuscitation due to life-limiting diagnoses were excluded. Data collection at each site was approved by either the institution’s research ethics board or quality improvement and data management committee.

### Data collection

Trained data abstractors were responsible for data acquisition in each hospital. Data were directly entered into a customized database with built-in error checking and a standard manual of operations and definitions. Data were electronically transmitted to the CHNN coordinating center in Children’s Hospital of Fudan University with patient identity kept confidential. Site investigators were responsible for data quality control at each site.

DR resuscitation was defined as receipt of any assistance after birth besides regular care––this includes providing warmth, drying and/or plastic wrap, and stimulation of the infant [4]. Infants were divided into five categories according to the highest intensity of resuscitation they received, in order from “lowest intensity” to “highest intensity”: (1) regular care including warmth, dry or plastic bag wrapping and suction when necessary; (2) oxygen supplementation and/or continuous positive airway pressure (O_2_/CPAP); (3) PPV via mask ventilation; (4) PPV via endotracheal intubation (ETT-PPV); and (5) CPR with or without medical therapy.

### Definitions

Intraventricular hemorrhage (IVH) was defined as ≥ grade 3 according to Papile’s criteria [10]. Cystic periventricular leukomalacia (cPVL) was defined as the presence of periventricular cysts on cranial ultrasound or magnetic resonance imaging. Severe brain injury was defined as IVH (grade ≥ 3) and cPVL. Early-onset sepsis was defined as sepsis occurring within 72 hours after birth. Late-onset sepsis was defined as sepsis occurring after 72 hours of age [11]. Respiratory distress syndrome (RDS) was diagnosed in preterm infants with the onset of respiratory distress shortly after birth and a compatible chest radiograph appearance [12]. GA was determined using the hierarchy of the best obstetric estimate based on prenatal first trimester dating ultrasound, menstrual history, obstetric examination, or all three. If the obstetric estimate was not available or was different from the postnatal estimate of gestation by more than two weeks, the GA was estimated using the Ballard score [13]. Small for gestational age (SGA) was defined as birth weight < 10th percentile for the GA according to the Chinese neonatal birth weight values [14]. Prenatal care was defined as ≥ 1 pregnancy-related hospital visit during pregnancy.

### Statistical analysis

Maternal and infant characteristics, as well as neonatal outcomes of infants, were compared using ANOVA or the Kruskal–Wallis test as appropriate for continuous variables and the Chi-square test for categorical variables. The trends of demographic characteristics and outcomes across resuscitation intensity levels were tested by the Cochrane–Armitage trend test for binary variables or the Jonckheere–Terpstra trend test for continuous variables. Univariate and inverse propensity score-weighted logistic regression models were generated to indicate the significance of neonatal outcomes and NICU resource use associated with the five intensity levels of DR resuscitation. Covariates for propensity score calculation are either tested significantly different across resuscitation intensity groups or investigator selected due to their clinical knowledge, including GA, SGA, inborn/outborn status, maternal age, maternal hypertension, premature rupture of membranes (PROM) > 24 hours, and antenatal steroids (ANS). A two-sided significance level of 0.05 was used without adjustment for multiple comparisons. All analyses were performed using SAS 9.4 software.

## Results

### Study population

**Fig. 1:**
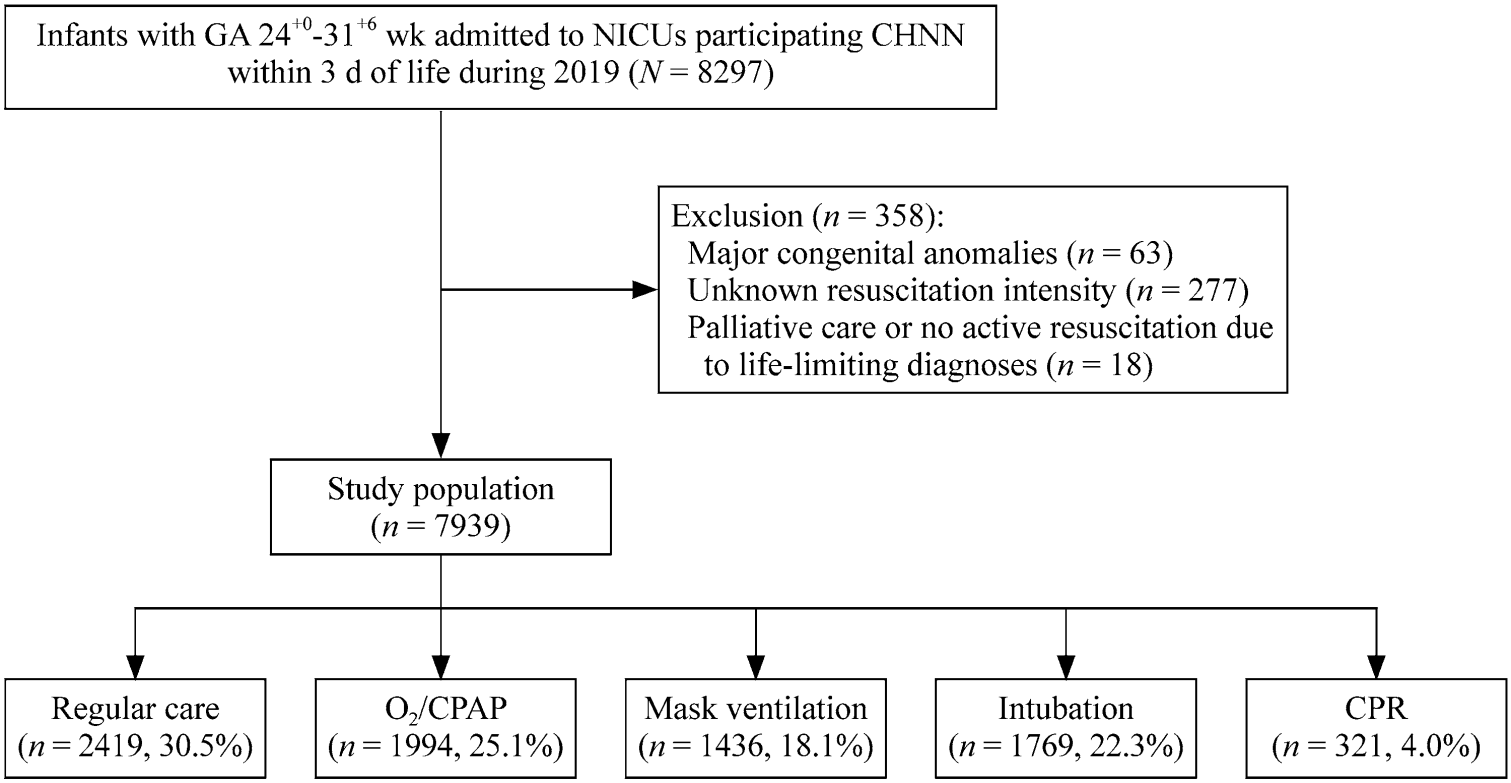
Flow chart for the study population. *GA* gestation age, *NICUs* neonatal intensive care units, *CHNN* Chinese Neonatal Network, *CPAP* continuous positive airway pressure, *CPR* cardiopulmonary resuscitation

A total of 8297 infants across forty-three centers were enrolled in the CHNN database during 2019 (Fig. 1). Sixty-three infants with major congenital anomalies, 277 with unknown resuscitation intensity, and 18 with palliative care or no resuscitation were excluded from this analysis.

### General population and variation across gestational age

**Fig. 2:**
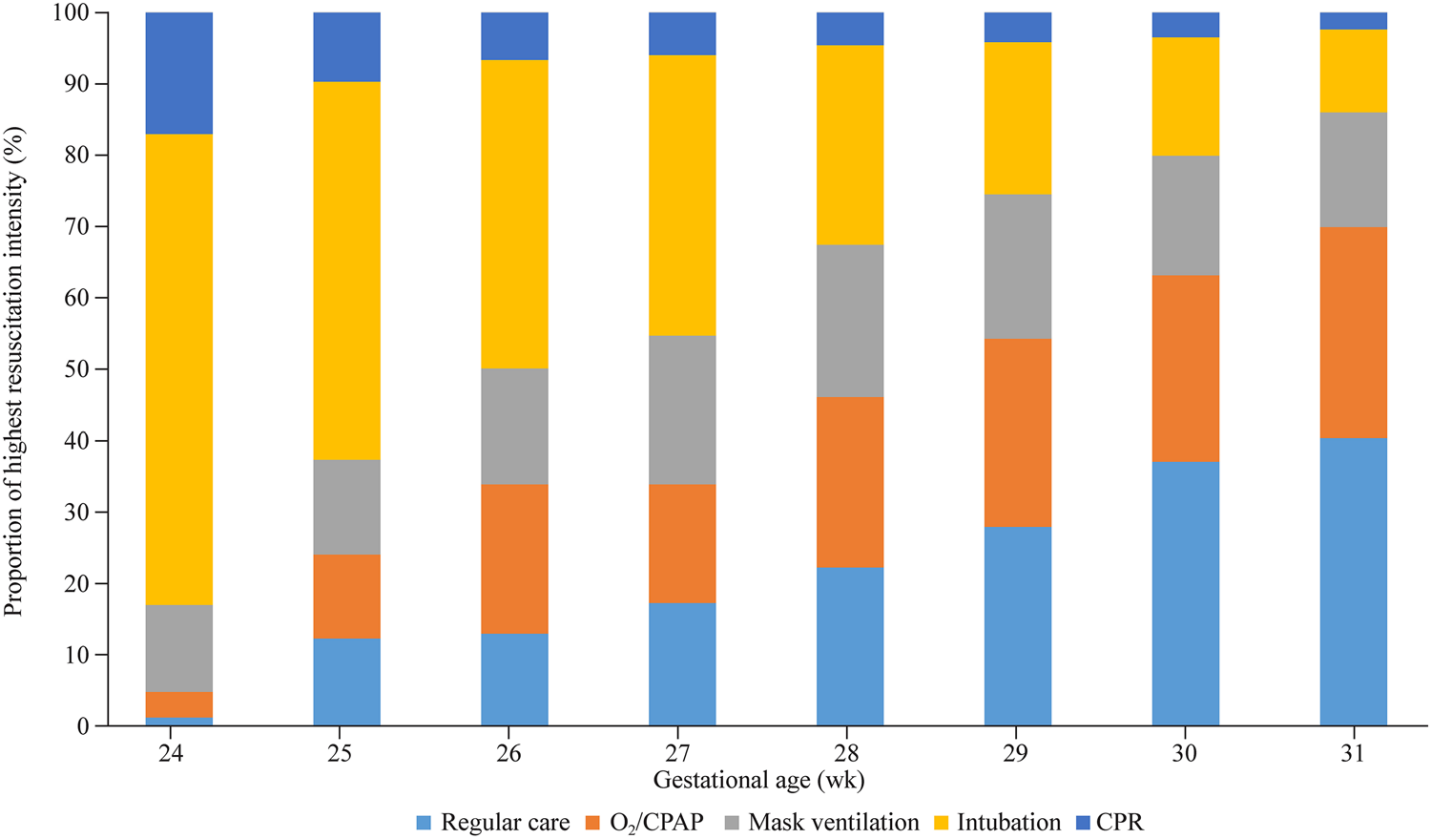
Variation in delivery room resuscitation intensity in China by gestational age of very preterm infants. The X-axis represents gestational age, and the Y-axis represents the proportion of each intensity of resuscitation. Different colors represent different resuscitation intensities. *CPAP* continuous positive airway pressure, *CPR* cardiopulmonary resuscitation

Of 7939 infants included in this cohort, 2419 (30.5%) received regular care without additional resuscitation; 1994 (25.1%) received O_2_/CPAP; 1436 (18.1%) received non-invasive mask ventilation; 1769 (22.3%) received ETT; and 321 (4.0%) were given CPR. The proportion of the different resuscitation intensities received at each GA is shown in Fig. 2. More than half of preterm infants at 24–25 weeks’ GA received endotracheal intubation in the DR, and with the increase in GA, the proportion of endotracheal intubation received gradually declined. Less than 25% of babies born after 29 weeks’ GA received ETT-PPV. In general, the older the GA, the fewer preterm infants receive endotracheal intubation or CPR.

### Variation across site

**Fig. 3:**
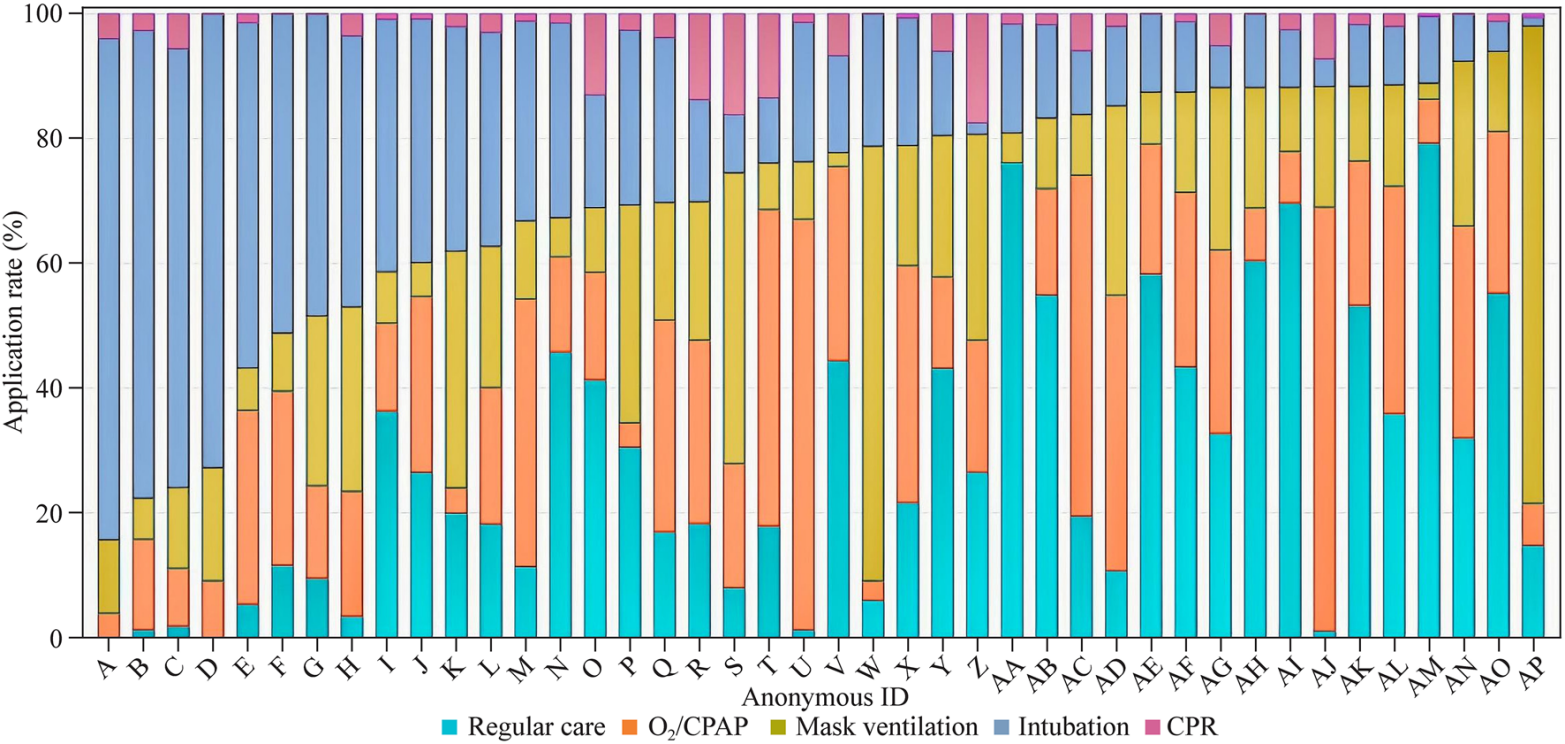
Variation in delivery room resuscitation intensity among very preterm infants between 24^+0^ and 31^+6^ weeks’ gestation in CHNN, emphasizing “inborn” infants. The X-axis represents the hospitals participating in this study, and the Y-axis represents the proportion of each intensity of resuscitation. Different colors represent different resuscitation intensities. They were ranked from high to low from left to right according to endotracheal intubation and CPR. *CHNN* Chinese Neonatal Network, *CPAP* continuous positive airway pressure, *CPR* cardiopulmonary resuscitation

The differences in the intensity of resuscitation between the different centers are shown in Fig. 3. Resuscitation strategies appear to vary widely among centers, with over 50% of preterm infants in eight (19%) centers requiring the highest intensity resuscitation (endotracheal intubation and CPR). There is also one center with more than 70% of preterm deliveries requiring mask ventilation. The resuscitation intensity in the remaining centers was mostly mild stimulation (CPAP and PPV via mask ventilation).

### Baseline characteristics

Maternal and neonatal characteristics are shown in Table 1. Lower birth weight and GA, lack of ANS, prolonged rupture of membranes, advanced maternal age, and inborn birth were associated with a higher intensity of resuscitation (*P* < 0.001). Maternal hypertension was significantly associated with a lower intensity of resuscitation. The lower the birth weight and GA, the greater the need for endotracheal intubation; preterm infants who require CPR have the lowest ANS use rate. As the incidence of PROM increases, the need for higher intensity resuscitation in the DR is lessened.

**Table 1:** Maternal and infant characteristics across resuscitation intensity group among very preterm infants between 24^+0^ and 31^+6^ weeks’ gestation in CHNN

Characteristics	Regular care (*n* = 2419)	O_2_/CPAP (*n* = 1994)	Mask ventilation (*n* = 1436)	Intubation (*n* = 1769)	CPR (*n* = 321)	*P*	*P* (trend)
Maternal characteristics							
Maternal age (y), mean (SD)	30.6 (4.9)	30.8 (5.0)	30.9 (4.8)	31.0 (4.8)	31.5 (4.9)	0.010	0.0001
Primigravida, *n*/*N* (%)	1233/2407 (51.2)	995/1980 (50.3)	776/1430 (54.3)	911/1750 (52.1)	156/320 (48.8)	0.150	–
Prenatal care, *n*/*N* (%)	2344/2375 (98.7)	1946/1962 (99.2)	1410/1424 (99.0)	1710/1724 (99.2)	299/306 (97.7)	0.090	–
Maternal diabetes, *n*/*N* (%)	427/2393 (17.8)	338/1982 (17.1)	268/1430 (18.7)	303/1740 (17.4)	55/319 (17.2)	0.770	–
Maternal hypertension, *n*/*N* (%)	433/2397 (18.1)	396/1980 (20.0)	260/1431 (18.2)	377/1745 (21.6)	76/319 (23.8)	< 0.010	< 0.010
PROM > 24 h, *n*/*N* (%)	658/2355 (27.9)	555/1931 (28.7)	323/1382 (23.4)	339/1664 (20.4)	60/296 (20.3)	< 0.001	< 0.001
ANS, *n*/*N* (%)	1922/2310 (83.2)	1528/1913 (79.9)	1087/1396 (77.9)	1221/1641 (74.4)	192/296 (64.9)	< 0.001	< 0.001
Cesarean delivery, *n*/*N* (%)	1348/2414 (55.8)	1156/1991 (58.1)	787/1435 (54.8)	1009/1758 (57.4)	193/321 (60.1)	0.190	–
Infant characteristics							
Gestational age (wk), median (IQR)	30.4 (29.3–31.1)	30.0 (29.0–31.0)	29.7 (28.4–30.9)	28.9 (27.6–30.1)	29.0 (27.7–30.3)	< 0.001	< 0.001
Birth weight (g), mean (SD)	1425.7 (297.9)	1367.1 (306.8)	1314.4 (301.3)	1194.5 (322.6)	1215.5 (326.9)	< 0.001	< 0.001
Small for gestational age, *n*/*N* (%)	153/2419 (6.3)	136/1994 (6.8)	99/1436 (6.9)	140/1769 (7.9)	21/321 (6.5)	0.390	–
Inborn, *n*/*N* (%)	1930/2419 (79.8)	1439/1994 (72.2)	1144/1436 (79.7)	1221/1769 (69.0)	201/321 (62.6)	< 0.001	< 0.001
Female, *n*/*N* (%)	1062/2415 (44.0)	864/1992 (43.4)	658/1435 (45.9)	769/1765 (43.6)	144/321 (44.9)	0.640	–
Multiple birth, *n*/*N* (%)	745/2419 (30.8)	583/1994 (29.2)	406/1436 (28.3)	571/1769 (32.3)	93/321 (29.0)	0.100	–

*CHNN* Chinese Neonatal Network, *PROM* premature rupture of membranes, *ANS* antenatal steroids, *CPAP* continuous positive airway pressure, *CPR* cardiopulmonary resuscitation, *IQR* interquartile range, *SD* standard deviation

### Observed incidence of outcomes across groups

Short-term outcomes among preterm infants with different levels of resuscitation are shown in Table 2. The incidence of overall death, early death (≤ 7 days old), severe brain impairment, IVH (grade ≥ 3), pneumothorax, and hypothermia on admission all increase significantly with the increase in the intensity of resuscitation. There was a trend of other adverse short-term outcomes increasing with higher levels of resuscitation, including sepsis, cPVL, and RDS. Because of the missing data because the infants died before the first ultrasound scan, the denominator of IVH ≥ grade 3 was lower than that of RDS or death.

**Table 2:** Observed rates of neonatal outcomes of very preterm infants between 24^+0^ and 31^+6^ weeks’ gestation in CHNN across resuscitation intensity group

Neonatal outcomes	Regular care (*n* = 2419)	O_2_/CPAP (*n* = 1994)	Mask ventilation (*n* = 1436)	Intubation (*n* = 1769)	CPR (*n* = 321)	*P* (trend)
Overall death	142/2419 (5.9)	145/1994 (7.3)	184/1436 (12.8)	374/1769 (21.1)	90/321 (28.0)	< 0.001
Early death (≤ 7 d)	89/2417 (3.7)	86/1990 (4.3)	108/1435 (7.5)	242/1766 (13.7)	64/319 (20.1)	< 0.001
Severe brain impairment	157/2078 (7.6)	159/1787 (8.9)	99/1239 (8.0)	237/1420 (16.7)	59/263 (22.4)	< 0.001
IVH grade ≥ 3	95/2067 (4.6)	89/1779 (5.0)	69/1235 (5.6)	170/1408 (12.1)	47/263 (17.9)	< 0.001
cPVL	88/2126 (4.1)	84/1828 (4.6)	51/1282 (4.0)	109/1485 (7.3)	16/274 (5.8)	< 0.010
Sepsis	187/2360 (7.9)	152/1928 (7.9)	147/1364 (10.8)	203/1626 (12.5)	27/282 (9.6)	< 0.001
Respiratory distress syndrome	1549/2418 (64.1)	1370/1992 (68.8)	1110/1433 (77.5)	1598/1766 (90.5)	279/321 (86.9)	< 0.001
Pneumothorax	18/2419 (0.7)	10/1994 (0.5)	15/1436 (1.0)	31/1769 (1.8)	8/321 (2.5)	< 0.001
Hypothermia on admission	1541/2409 (64.0)	1423/1986 (71.7)	946/1426 (66.3)	1196/1757 (68.1)	241/319 (75.6)	< 0.010

Data are presented as *n*/*N* (%). *CHNN* Chinese Neonatal Network, *CPAP* continuous positive airway pressure, *CPR* cardiopulmonary resuscitation, *IVH* intraventricular hemorrhage, *cPVL* cystic periventricular leukomalacia

### The odds ratios between resuscitation intensity and incidence of outcomes

After adjusting for GA, SGA, inborn/outborn status, maternal age, maternal hypertension, PROM > 24 hours, and ANS, multivariate regression analysis showed that the risk of early death and overall death gradually increased with the intensity of resuscitation. CPR greatly increased the risk of early death and overall death. Sepsis and cPVL were highly associated with endotracheal intubation; RDS was highly associated with endotracheal intubation and CPR; IVH (grade ≥ 3) and pneumothorax were related to CPR (Table 3).

**Table 3:** Inverse propensity score-weighted adjusted OR^a, b^ (95% CI) of neonatal outcomes across resuscitation intensity group among very preterm infants between 24^+0^ and 31^+6^ weeks’ gestation in CHNN

Neonatal outcomes	O_2_/CPAP	Mask ventilation	Intubation	CPR
Overall death	0.95 (0.76, 1.18)	1.46 (1.17, 1.82)	2.02 (1.66, 2.46)	3.36 (2.48, 4.54)
Early death (≤ 7 d)	0.95 (0.73, 1.25)	1.31 (1.00, 1.73)	1.99 (1.56, 2.52)	4.04 (2.88, 5.67)
Severe brain impairment	1.01 (0.80, 1.28)	0.89 (0.68, 1.17)	1.70 (1.36, 2.12)	2.37 (1.64, 3.43)
IVH grade ≥ 3	0.92 (0.68, 1.24)	0.99 (0.72, 1.37)	1.86 (1.42, 2.44)	3.04 (2.02, 4.59)
cPVL	0.96 (0.70, 1.32)	0.87 (0.60, 1.24)	1.43 (1.05, 1.93)	0.99 (0.52, 1.89)
Sepsis	0.90 (0.72, 1.12)	1.13 (0.90, 1.43)	1.32 (1.07, 1.63)	0.91 (0.58, 1.44)
Respiratory distress syndrome	1.08 (0.95, 1.24)	1.42 (1.22, 1.66)	3.27 (2.75, 3.89)	2.40 (1.74, 3.32)
Pneumothorax	0.58 (0.25, 1.33)	1.37 (0.68, 2.79)	1.85 (0.99, 3.46)	4.14 (1.85, 9.27)
Hypothermia on admission	1.32 (1.16, 1.51)	0.99 (0.86, 1.15)	1.02 (0.89, 1.17)	1.76 (1.32, 2.35)

*CHNN* Chinese Neonatal Network, *CPAP* continuous positive airway pressure, *CPR* cardiopulmonary resuscitation, *IVH* intraventricular hemorrhage, *cPVL* cystic periventricular leukomalacia, *OR* odds ratio, *CI* confidence interval, *GA* gestation age, *SGA* small for gestational age, *PROM* premature rupture of membranes, *ANS* antenatal steroids. ^a^Regular care as the reference group; ^b^adjusted for variables tested significant in Table 1 or investigator selected, including GA, SGA, inborn/outborn status, maternal age, maternal hypertension, PROM > 24 hour and ANS

### Intensive resuscitation and different gestational ages

Infants were divided into either no intensive resuscitation or intensive resuscitation groups depending on whether they received intubation or CPR (Supplementary Table 1). After adjusting for perinatal factors, including GA, SGA, inborn/outborn status, maternal age, maternal hypertension, PROM > 24 hours, and ANS, multivariate regression analysis showed that short-term outcomes, such as overall death [adjusted odds ratio (aOR) = 1.54, 95% confidence interval (CI) = 1.31–1.82], early death (≤ 7 days old) (aOR = 1.57, 95% CI = 1.28–1.91), severe brain impairment (aOR = 1.83, 95% CI = 1.48–2.26), IVH grade ≥ 3 (aOR = 1.99, 95% CI = 1.55–2.55), cPVL (aOR = 1.53, 95% CI = 1.13–2.06), RDS (aOR = 2.62, 95% CI = 2.16–3.17), and pneumothorax (aOR 2.05, 95% CI = 1.15–3.65), were at increased risk of occurring after intensive resuscitation.

The profiles of extremely preterm infants (24–27^+6^) and very preterm infants (28–31^+6^) are shown in Supplementary Tables 2 and 3. Comparing the effects of ventilation of very preterm infants and extremely preterm infants on the outcome shows that extremely preterm infants have a higher proportion of intensive resuscitation for different outcomes, such as overall death or early death and severe brain impairment (IVH grade ≥ 3, cPVL).

## Discussion

### Resuscitation status and variation in resuscitation intensity across the country

In this research, by understanding the variability of resuscitation across centers, a baseline was obtained to provide a basis for future quality improvement. We will adopt homogeneous management and training to narrow this difference. Our research reveals differences in the intensity of resuscitation of preterm infants at 24^+0^–31^+6^ weeks of GA in China and provides useful data for resuscitation practices in the DR for extremely preterm infants and very preterm infants. A study by Bajaj et al. showed that 76% of 29^+0^–33^+6^ weeks’ preterm infants received a certain degree of resuscitation in the DR, 14.7% received endotracheal intubation, and 2.7% received CPR [4]; in a study in South Korea, nearly 93% of infants received supplemental oxygen at 29–32 weeks’ GA, and 3% received CPR in the DR [15]. Boyle et al. reported that 36.9% of infants delivered at 32–33 weeks’ GA received active resuscitation in the DR [16]. A multicenter study in northern China showed that the proportion of preterm infants less than 28 weeks of GA and less than 1000 g of birth weight who received endotracheal intubation was 69.5%, and the proportion receiving chest compressions was 18.2% [7]. A study on the morbidity and mortality of preterm infants < 32 weeks of GA in China showed that 26.7% received endotracheal intubation [9]. In our study, 69.5% of preterm infants at 24^+0^–31^+6^ weeks received resuscitation in the DR and 22.3% required ETT-PPV without CPR and an additional 4% required CPR, which is higher than studies from other countries and lower than other studies completed in China. However, studies from other countries tended to focus on very preterm infants (and did not include extremely preterm infants). Our research has confirmed the difference in the intensity of resuscitation received for extremely preterm infants and very preterm infants.

In our study, we described that the lower the GA, the higher the intensity of resuscitation needed. When GA was less than 26 weeks, more than 50% of preterm infants received a higher intensity of resuscitation, including endotracheal intubation and CPR. It has been shown in other studies that half of preterm infants at 24 weeks of GA may be able to maintain respiratory stability on DR-CPAP [17]. In our study, the proportion stabilized on DR-CPAP was lower (25.1% vs. 32.5%) [4].

Our study emphasizes the variation in the intensity of resuscitation of preterm infants “inborn” less than 32 weeks of GA; 26.3% of preterm infants received higher intensity resuscitation, such as endotracheal intubation. Our study includes data from 57 hospitals in 25 provinces across the country. A multicenter study in northern China (including tertiary and secondary hospitals in five provinces and regions) showed that the proportion of preterm infants less than 28 weeks of GA receiving high-intensity resuscitation, such as endotracheal intubation, was 68.4% [7]. There are obvious differences in the intensity of resuscitation practices in different regions and different hospitals in China. This reminds us that we should carry out targeted training and quality improvement to improve the standardization of resuscitation practices for extremely premature infants and very preterm infants.

### Higher intensity of resuscitation results in higher rates of mortality and morbidities

There is a correlation between the mortality of preterm infants and the intensity of resuscitation. The mortality rate of preterm infants in our study was 11.8%, which is lower than previously reported rates in China [18, 19] and similar to the report of Lee et al. in South Korea [20]. It is still higher than the 7.4% reported by the Canadian Neonatal Network in 2018 [5]. We found that higher mortality rates are associated with preterm infants who received CPR [21–23]. As the intensity of resuscitation increases, the mortality rate increases. Our study shows that mortality after CPR is higher than that in the study by Bajaj et al. (28% vs. 21%), and the risk of death after CPR is significantly higher than that of other resuscitative measures, especially in the first week of life. Low-intensity resuscitation had a significantly lower mortality rate (CPR 20.1% vs. O_2_/CPAP 4.3%). Research by Arnon et al. showed that CPR is an important predictor of death or adverse outcomes [24].

Some studies have shown that severe morbidity (IVH ≥ grade 3 or cPVL) was higher in the CPR group [3, 4, 21, 24]. Our study demonstrated that DR intubation and CPR were associated with a higher risk of severe IVH among very preterm infants between 24^+0^ and 31^+6^ weeks’ gestation. This finding is also consistent with the large cohort study by Handley et al. [23]. In our univariate analysis, we found higher rates of cPVL and sepsis in the CPR group. However, this difference was not significant after adjustment for confounders. This is probably because the neonates who died in the DR were not recorded in the database, causing a bias in the results.

In Table 2, the rates of hypothermia on admission were extremely high, but there was no significant difference. This means that insulation did not perform effectively during the resuscitation process, which we will enhance in future quality improvements. ANS can accelerate fetal lung development and reduce morbidity and mortality in preterm infants between 22 and 34 weeks of GA [17, 25], as well as reduce extensive resuscitation [26]. In this research, the use of ANS was 83.2% in preterm infants without resuscitation compared with 64.9% who needed CPR, indicating that ANS reduced the intensity of resuscitation. Identification of high-risk pregnancies will help pediatricians prepare for resuscitation more fully.

### Strengths and limitations

The first strength of this study was that we enrolled a very large population of very preterm infants between 24^+0^ and 31^+6^ weeks’ gestation in China. Second, the data collected were rigorously assessed in detail, especially DR resuscitation data. Third, we included all “inborn” infants from fifty-seven hospitals—forty-three hospitals participated in this study, which are widely distributed throughout China, giving a broad overview of resuscitative practices.

This study has some limitations. First, as a retrospective cross-sectional study, it is impossible to trace the specific indications for resuscitation, such as intubation and chest compressions in CPR. Second, this study is an observational study and may not show the impact of interventions on the long-term outcome of preterm infants. Third, because some cases with incomplete data were excluded and the neonates who died in the DR were not recorded in the database, there may be bias in the results. Finally, several differences in DR management of preterm babies in NICUs may affect the final results.

In conclusion, the intensity of resuscitation is associated with an increased risk of death or severe brain injury. The variation in resuscitative practices in different hospitals across China is evident, and to carry out quality improvement and achieve standardized resuscitation, we will use theoretical research, simulation training, and other techniques.

### Supplementary Information

Below is the link to the electronic supplementary material.

Supplementary file 1 (PDF 82 KB)

Supplementary file 2 (PDF 124 KB)

Supplementary file 3 (MP4 219679 KB)

## Data Availability

Data will be made available on reasonable request.

## References

[1] Aziz K, Lee CHC, Escobedo MB, Hoover AV, Kamath-Rayne BD, Kapadia VS (2021). Part 5: neonatal resuscitation 2020 American Heart Association guidelines for cardiopulmonary resuscitation and emergency cardiovascular care. Pediatrics.

[2] Wyckoff MH, Wyllie J, Aziz K, de Almeida MF, Fabres JW, Fawke J (2020). Neonatal life support 2020 international consensus on cardiopulmonary resuscitation and emergency cardiovascular care science with treatment recommendations. Resuscitation.

[3] Fischer N, Soraisham A, Shah PS, Synnes A, Rabi Y, Singhal N (2019). Extensive cardiopulmonary resuscitation of preterm neonates at birth and mortality and developmental outcomes. Resuscitation.

[4] Textbook of neonatal resuscitation. 8th ed. Elk Grove Village: American Academy of Pediatrics; 2021

[5] Beltempo M, Shah P, Yoon EW, Chan P, Balachandran N, Members of the Annual Report Review Committee. Canadian Neonatal Network 2018 annual report. Toronto, Ontario: Canadian Neonatal Network; 2018.

[6] Beltempo M, Isayama T, Vento M, Lui K, Kusuda S, Lehtonen L (2018). Respiratory management of extremely preterm infants: an international survey. Neonatology.

[7] Li SJ, Feng Q, Tian XY, Zhou Y, Ji Y, Li YM (2021). Delivery room resuscitation and short-term outcomes of extremely preterm and extremely low birth weight infants: a multicenter survey in North China. Chin Med J (Engl).

[8] Force CNRPT (2016). China neonatal resuscitation guideline (revised in 2016, Beijing). Chin J Perinat Med.

[9] Cao Y, Jiang S, Sun J, Hei M, Wang L, Zhang H (2021). Assessment of neonatal intensive care unit practices, morbidity, and mortality among very preterm infants in China. JAMA Netw Open.

[10] Papile LA, Burstein J, Burstein R, Koffler H (1978). Incidence and evolution of subependymal and hemorrhage: a study of infants with birth weights less than 1500 gm. J Pediatr.

[11] Stoll BJ, Hansen N, Fanaroff AA, Wright LL, Carlo WA, Ehrenkranz RA (2002). Late-onset sepsis in very low birth weight neonates: the experience of the nichd neonatal research network. Pediatrics.

[12] Bhakta K (2012). Respiratory distress syndrome. Manual of neonatal care.

[13] Ballard JL, Novak KK, Driver M (1979). A simplified score for assessment of fetal maturation of newly born infants. J Pediatr.

[14] Zhu L, Zhang R, Zhang S, Shi W, Yan W, Wang X (2015). Chinese neonatal birth weight curve for different gestational age. Zhonghua Er Ke Za Zhi.

[15] Cho SJ, Shin J, Namgung R (2015). Initial resuscitation at delivery and short term neonatal outcomes in very-low-birth-weight infants. J Korean Med Sci.

[16] Boyle EM, Johnson S, Manktelow B, Seaton SE, Draper ES, Smith LK (2015). Neonatal outcomes and delivery of care for infants born late preterm or moderately preterm: a prospective population-based study. Arch Dis Child Fetal Neonatal Ed.

[17] Patel PN, Banerjee J, Godambe SV (2016). Resuscitation of extremely preterm infants—controversies and current evidence. World J Clin Pediatr.

[18] Wu F, Liu G, Feng Z, Tan X, Yang C, Ye X (2019). Short-term outcomes of extremely preterm infants at discharge: a multicenter study from Guangdong province during 2008–2017. BMC Pediatr.

[19] Jiang S, Yan W, Li S, Zhang L, Zhang Y, Shah PS (2020). Mortality and morbidity in infants <34 weeks’ gestation in 25 NICUs in China: a prospective cohort study. Front Pediatr.

[20] Lee JH, Noh OK, Chang YS, Korean Neonatal Network (2019). Neonatal outcomes of very low birth weight infants in Korean Neonatal Network from 2013 to 2016. J Korean Med Sci.

[21] Shukla V, Elkhateeb O, Shah PS, Yang J, Lee KS, Canadian Neonatal Network Investigators (2020). Outcomes of neonates born at 26 weeks gestational age who receive extensive cardiopulmonary resuscitation compared with airway and breathing support. J Perinatol.

[22] Soraisham AS, Lodha AK, Singhal N, Aziz K, Yang J, Lee SK (2014). Neonatal outcomes following extensive cardiopulmonary resuscitation in the delivery room for infants born at less than 33 weeks gestational age. Resuscitation.

[23] Handley SC, Sun Y, Wyckoff MH, Lee HC (2015). Outcomes of extremely preterm infants after delivery room cardiopulmonary resuscitation in a population-based cohort. J Perinatol.

[24] Arnon S, Dolfin T, Reichman B, Regev RH, Geva LL, Boyko V (2017). Delivery room resuscitation and adverse outcomes among very low birth weight preterm infants. J Perinatol.

[25] Travers CP, Carlo WA, Mcdonald SA, Das A, Bell EF, Ambalavanan N (2018). Mortality and pulmonary outcomes of extremely preterm infants exposed to antenatal corticosteroids. Am J Obstet Gynecol.

[26] Lee J, Lee JH (2019). A clinical scoring system to predict the need for extensive resuscitation at birth in very low birth weight infants. BMC Pediatr.

