# Supplementary material for: Delivery room resuscitation intensity and associated neonatal outcomes of 24+0–31+6 weeks’ preterm infants in China: a retrospective cross-sectional study
Source: World J Pediatr. 2023 Jun 30;20(1):64–72. doi: 10.1007/s12519-023-00738-2 (PMC10827838; doi:10.1007/s12519-023-00738-2)
Supplement: Supplementary file 1 — (PDF 82 KB) [file 12519_2023_738_MOESM1_ESM.pdf]

Group information of the Chinese Neonatal Network:

Chairmen: Shoo K. Lee[8], Mount Sinai Hospital, University of Toronto; Chao Chen[9], Children's Hospital of Fudan University. Vice-Chairmen: Wen-Hao Zhou[10], Children's Hospital of Fudan University. Site principle investigators of the Chinese Neonatal Network: Fa-Lin Xu[11], The Third Affiliated Hospital of Zhengzhou University; Xiu-Ying Tian[12], Tianjin Obstetrics & Gynecology Hospital; Hua-Yan Zhang[13], Guangzhou Women and Children's Medical Center; Yong Ji[14], Children's Hospital of Shanxi; Zhan-Kui Li[15], Northwest Women's and Children's Hospital; Jing-Yun Shi[16], Gansu Provincial Maternity and Child Care Hospital; Xin-Dong Xue[17], Shengjing Hospital of China Medical University; Dong-Mei Chen[18], Quanzhou Women and Children's Hospital; San-Nan Wang[19], The Affiliated Suzhou Hospital of Nanjing Medical University; Ling Liu[20], Guizhou Women and Children's Hospital/Guiyang Children's Hospital; Xi-Rong Gao[21], Hunan Children's Hospital; Hui Wu[22], The First Bethune Hospital of Jilin University; Chang-Yi Yang[23], Fujian Maternity and Child Health Hospital, Affiliated Hospital of Fujian Medical University; Shu-Ping Han[24], Nanjing Maternity and Child Health Care Hospital; Ruo-Bing Shan[25], Qingdao Women and Children's Hospital; Hong Jiang[26], The Affiliated Hospital of Qingdao University; Gang Qiu[27], Children's Hospital of Shanghai; Qiu-Fen Wei[28], Women and Children's Hospital of Guangxi Zhuang Autonomous Region; Rui Cheng[29], Children's Hospital of Nanjing Medical University; Wen-Qing Kang[30], Henan Children's Hospital; Ming-Xia Li[31], The First Affiliated Hospital of Xinjiang Medical University; Yi-Heng Dai[32], Foshan Women and Children's Hospital; Li-Li Wang[33], The First Affiliated Hospital of Anhui Medical University; Zhen-Lang Lin[34], Yuying Children's Hospital Affiliated to Wenzhou Medical University;

Yuan Shi[35], Children’s Hospital of Chongqing Medical University; Xiu-Yong Cheng[36], The First Affiliated Hospital of Zhengzhou University; Jia-Hua Pan[37], The First Affiliated Hospital of USTC, Division of Life Sciences and Medicine, University of Science and Technology of China; Qin Zhang[38], Shaanxi Provincial People’s Hospital; Xing Feng[39], Children's Hospital of Soochow University; Qin Zhou[40], Wuxi Maternity and Child Healthcare Hospital; Long Li[41], People's Hospital of Xinjiang Uygur Autonomous Region; Ping-Yang Chen[42], The Second Xiangya Hospital of Central South University; Xiao-Ying Li[43], Qilu Children’s Hospital of Shandong University; Ling Yang[44], Hainan Women and Children’s Hospital; De-Yi Zhuang[45], Xiamen Children’s Hospital; Yong-Jun Zhang[46], Xinhua Hospital affiliated to Shanghai Jiao Tong University School of Medicine; Jin-Xing Feng[47], Shenzhen Children’s Hospital; Li Li[48], Children's Hospital Affiliated to Capital Institute of Pediatrics; Xin-Zhu Lin[49], Women and Children’s Hospital, School of Medicine, Xiamen university; Yin-Ping Qiu[50], General Hospital of Ningxia Medical University; Kun Liang[51], First Affiliated Hospital of Kunming Medical University; Li Ma[52], Hebei Provincial Children's Hospital; Li-Ping Chen[53], Jiangxi Provincial Children’s Hospital; Li-Yan Zhang[54], Fuzhou Children’s Hospital of Fujian Province; Hong-Xia Song[55], First Affiliated Hospital of Xian Jiao Tong University; Ming-Yan Hei[56], Beijing Children's Hospital, Capital Medical University; Hui-Wen Huang[57], Zhuhai Center for Maternal and Child Health Care; Jie Yang[58], Guangdong Women and Children's Hospital; Dong Li[59], Dalian Municipal Women and Children’s Medical Center; Guo-Fang Ding[60], Peking Union Medical College Hospital; Ji-Mei Wang[61], Obstetrics & Gynecology Hospital of Fudan University; Qian-Shen Zhang[62], Shenzhen Hospital of Hongkong University; Xiao-Lu Ma[63], Children's Hospital of Zhejiang

University School of Medicine. Advisor: Joseph Ting[64], University of Alberta.
