# Supplementary material for: Delivery room resuscitation intensity and associated neonatal outcomes of 24+0–31+6 weeks’ preterm infants in China: a retrospective cross-sectional study
Source: World J Pediatr. 2023 Jun 30;20(1):64–72. doi: 10.1007/s12519-023-00738-2 (PMC10827838; doi:10.1007/s12519-023-00738-2)
Supplement: Supplementary file 2 — (PDF 124 KB) [file 12519_2023_738_MOESM2_ESM.pdf]

Supplementary Table 1. Observed rates and adjusted OR[a, b] (95% CI) of neonatal outcomes between the groups with or without intensive resuscitation among very preterm infants between 24^{+0}-31^{+6} weeks' gestation in CHNN

**Neonatal outcomes**	**Intensive resuscitation**		**OR (95% CI)**	
	**No (*N* = 5849)**	**Yes (*N* = 2090)**	**Crude[a]**	**Adjusted[a, b]**
Overall death	471/5849 (8.1)	464/2090 (22.2)	3.26 (2.83, 3.75)	1.54 (1.31, 1.82)
Early death (≤ 7 d)	283/5842 (4.8)	306/2085 (14.7)	3.38 (2.85, 4.01)	1.57 (1.28, 1.91)
Severe brain impairment	415/5104 (8.1)	296/1683 (17.6)	2.41 (2.05, 2.83)	1.83 (1.48, 2.26)
IVH grade ≥ 3	253/5081 (5.0)	217/1671 (13.0)	2.85 (2.35, 3.45)	1.99 (1.55, 2.55)
cPVL	223/5236 (4.3)	125/1759 (7.1)	1.72 (1.37, 2.16)	1.53 (1.13, 2.06)
Sepsis	486/5652 (8.6)	230/1908 (12.1)	1.46 (1.23, 1.72)	1.14 (0.93, 1.40)
Respiratory distress syndrome	4029/5843 (69.0)	1877/2087 (89.9)	4.02 (3.45, 4.69)	2.62 (2.16, 3.17)
Pneumothorax	43/5849 (0.7)	39/2090 (1.9)	2.57 (1.66, 3.97)	2.05 (1.15, 3.65)
Hypothermia on admission	3910/5821 (67.2)	1437/2076 (69.2)	1.10 (0.99, 1.23)	0.97 (0.84, 1.11)

CHNN Chinese Neonatal Network, *IVH* intraventricular hemorrhage, *cPVL* cystic periventricular leukomalacia, *OR* odds ratio, *CI* confidence interval, *GA* gestation age, *SGA* small-for-gestational age, *PROM* premature rupture of membranes, *ANS* antenatal steroids, *CPR* cardiopulmonary resuscitation. [a]"No intensive resuscitation" as the reference group, while intensive resuscitation refers to intubation and/or CPR; [b]adjusted for variables tested significant in Table 1 or investigator selected, including GA, SGA, inborn/outborn status, maternal age, maternal hypertension, PROM > 24 h and ANS

Supplementary Table 2. Observed rates and adjusted OR[a, b] (95% CI) of neonatal outcomes between the groups with or without intensive resuscitation among very preterm infants between 28^{+0}-31^{+6} weeks' gestation in CHNN

Neonatal outcomes	Intensive resuscitation		OR (95% CI)	
	**No (*N* = 5228)**	**Yes (*N* = 1432)**	**Crude[a]**	**Adjusted[a, b]**
Overall death	299/5228 (5.7)	216/1432 (15.1)	2.93 (2.43, 3.53)	2.00 (1.55, 2.56)
Early death (≤ 7 d)	179/5221 (3.4)	150/1431 (10.5)	3.30 (2.63, 4.13)	2.14 (1.58, 2.90)
Severe brain impairment	337/4603 (7.3)	172/1171 (14.7)	2.18 (1.79, 2.65)	1.85 (1.40, 2.44)
IVH grade ≥ 3	192/4580 (4.2)	119/1160 (10.3)	2.61 (2.06, 3.32)	2.14 (1.52, 3.02)
cPVL	193/4713 (4.1)	77/1227 (6.3)	1.57 (1.20, 2.06)	1.48 (1.01, 2.17)
Sepsis	404/5101 (7.9)	144/1345 (10.7)	1.39 (1.14, 1.70)	1.22 (0.94, 1.59)
Respiratory distress syndrome	3470/5224 (66.4)	1248/1429 (87.3)	3.49 (2.95, 4.12)	2.80 (2.27, 3.45)
Pneumothorax	34/5228 (0.7)	26/1432 (1.8)	2.83 (1.69, 4.72)	2.65 (1.22, 5.80)
Hypothermia on admission	3479/5208 (66.8)	954/1421 (67.1)	1.02 (0.90, 1.15)	0.93 (0.78, 1.10)

CHNN Chinese Neonatal Network, *IVH* intraventricular hemorrhage, *cPVL* cystic periventricular leukomalacia, *OR* odds ratio, *CI* confidence interval, *GA* gestation age, *SGA* small-for-gestational age, *PROM* premature rupture of membranes, *ANS* antenatal steroids, *CPR* cardiopulmonary resuscitation. [a]"No intensive resuscitation" as the reference group, while intensive resuscitation refers to intubation and/or CPR; [b]adjusted for variables tested significant in Table 1 or investigator selected, including GA, SGA, inborn/outborn status, maternal age, maternal hypertension, PROM > 24 h and ANS

Supplementary Table 3. Observed rates and adjusted OR[a, b] (95% CI) of neonatal outcomes between the groups with or without intensive resuscitation among very preterm infants between 24^{+0}-27^{+6} weeks' gestation in CHNN

**Neonatal outcomes**	**Intensive resuscitation**		**OR (95% CI)**	
	**No (*N* = 5228)**	**Yes (*N* = 1432)**	**Crude[a]**	**Adjusted[a, b]**
Overall death	299/5228 (5.7)	216/1432 (15.1)	2.93 (2.43, 3.53)	2.00 (1.55, 2.56)
Early death (≤ 7 d)	179/5221 (3.4)	150/1431 (10.5)	3.30 (2.63, 4.13)	2.14 (1.58, 2.90)
Severe brain impairment	337/4603 (7.3)	172/1171 (14.7)	2.18 (1.79, 2.65)	1.85 (1.40, 2.44)
IVH grade ≥ 3	192/4580 (4.2)	119/1160 (10.3)	2.61 (2.06, 3.32)	2.14 (1.52, 3.02)
cPVL	193/4713 (4.1)	77/1227 (6.3)	1.57 (1.20, 2.06)	1.48 (1.01, 2.17)
Sepsis	404/5101 (7.9)	144/1345 (10.7)	1.39 (1.14, 1.70)	1.22 (0.94, 1.59)
Respiratory distress syndrome	3470/5224 (66.4)	1248/1429 (87.3)	3.49 (2.95, 4.12)	2.80 (2.27, 3.45)
Pneumothorax	34/5228 (0.7)	26/1432 (1.8)	2.83 (1.69, 4.72)	2.65 (1.22, 5.80)
Hypothermia on admission	3479/5208 (66.8)	954/1421 (67.1)	1.02 (0.90, 1.15)	0.93 (0.78, 1.10)

CHNN Chinese Neonatal Network, *IVH* intraventricular hemorrhage, *cPVL* cystic periventricular leukomalacia, *OR* odds ratio, *CI* confidence interval, *GA* gestation age, *SGA* small-for-gestational age, *PROM* premature rupture of membranes, *ANS* antenatal steroids, *CPR* cardiopulmonary resuscitation. [a]"No intensive resuscitation" as the reference group, while intensive resuscitation refers to intubation and/or CPR; [b]adjusted for variables tested significant in Table 1 or investigator selected, including GA, SGA, inborn/outborn status, maternal age, maternal hypertension, PROM > 24 h and ANS
